# From Curiosity to Consumption: Consumer Attitudes Toward Alternative Proteins in Northwestern Italy

**DOI:** 10.3390/foods14213727

**Published:** 2025-10-30

**Authors:** Aitor Garcia-Vozmediano, Carla Ferraris, Giovanna Gallo, Cecilia Guasco, Alessandra Provera, Silvia Olivieri, Giulia Scardino, Fabio Zuccon, Monica Pitti, Daniela Manila Bianchi, Marco Savino Di Trani, Cristiana Maurella

**Affiliations:** 1 Istituto Zooprofilattico Sperimentale del Piemonte Liguria e Valle d’Aosta, Via Bologna 148, 10154 Turin, Italy; aitor.garciavozmediano@izsplv.it (A.G.-V.); giovanna.gallo@izsplv.it (G.G.); cecilia.guasco@izsplv.it (C.G.); alessandra.provera@izsplv.it (A.P.); silvia.olivieri@izsplv.it (S.O.); giulia.scardino@asl.novara.it (G.S.); fabio.zuccon@izsplv.it (F.Z.); monica.pitti@izsplv.it (M.P.); manila.bianchi@izsplv.it (D.M.B.); savinomarco.ditrani@izsplv.it (M.S.D.T.); cristiana.maurella@izsplv.it (C.M.); 2 Centro di Referenza Nazionale per la Rilevazione negli Alimenti di Sostanze e Prodotti che Provocano Allergie e Intolleranze—CReNaRiA, Via Bologna 148, 10154 Turin, Italy; 3 Centro di Referenza Regionale per la Sorveglianza sulle Patologie delle Chiocciole, la Salubrità dei Prodotti Alimentari Derivati e la Sostenibilità Ambientale—CeLI, Via Sandro Pertini 11, 12100 Cuneo, Italy

**Keywords:** alternative proteins, plant-based alternatives, edible insects, consumer acceptance, sustainable diets

## Abstract

Consumer interest in alternative protein sources has grown rapidly, driven by concerns over health, sustainability and environmental impact. This study investigated perceptions, attitudes and behaviours towards alternative proteins among residents of Piedmont, northwestern Italy. A cross-sectional online and face-to-face survey was conducted between August and October 2023, collecting sociodemographic data, dietary habits, and respondents’ perception on plant-, insect-, snail-, and algae-derived products. Responses from 627 participants were analysed. Approximately one-third reported consuming alternative proteins, with uptake being higher among women, younger adults, and flexitarian respondents. Plant-based alternatives were by far the most frequently consumed, while insect- and snail-based products elicited strong aversion. Curiosity and interest on alternative options predominated among consumers, whereas indifference and disgust—particularly among women and towards animal-derived options—were common among non-consumers. Reported barriers included taste, quality, and safety concerns, with price emerging as a major constraint. These findings indicate that acceptance of alternative proteins is unevenly distributed across demographic groups and product types. Plant-based options currently appear best positioned to facilitate dietary shifts, whereas animal-derived alternatives remain hindered by cultural and sensory barriers. Supporting the protein transition will require strategies to improve affordability, sensory appeal and consumer trust.

## 1. Introduction

The global population is projected to reach 9.8 billion by 2050 [1]. This growth is expected to lead to a significant increase in food demand, placing additional pressure on agricultural systems and natural resources, which are already stressed by climate change [2,3]. A substantial share of this demand will concern proteins of both animal and plant origin, highlighting the need for sustainable alternatives that can meet future nutritional requirements without exceeding planetary boundaries [4].

In parallel with environmental and demographic pressures, consumer awareness of animal welfare has risen considerably [5]. This has driven shifts in consumption patterns, as growing numbers of individuals seek ethically produced foods [6]. Consequently, alternative protein sources are gaining popularity as ethical options, supporting the expansion of consumer groups such as vegetarians, vegans, and flexitarians [7].

Proteins play a central role in human health, contributing to cellular regeneration, immune regulation, and the preservation of muscle mass [8]. However, the food industry faces increasing pressure to ensure both food safety and nutritional adequacy, as global protein rises in line with urbanisation and economic growth [9]. These challenges have stimulated the development and promotion of alternative protein sources, including plant-based options such as soy, lentils, chickpeas, and algae, as well as microbial proteins and insects [10]. These sources are increasingly recognised as promising strategies for reducing environmental impacts and enhancing the resilience of food systems [11].

In response, a range of innovative solutions has emerged, including cultured meat, single-cell proteins, vertical farming systems and novel plant- and animal-based protein sources [12]. Such technologies are positioned as ways of addressing the environmental and ethical challenges of protein production and they align closely with international policy frameworks such as the Paris Agreement and the United Nations’ Sustainable Development Goals [13,14].

Despite growing public interest in alternative proteins, consumer acceptance remains a critical factor market integration [15]. Increased familiarity with production processes and emerging technologies has not always translated into consumer confidence, and this remains a major challenge, as consumer scepticism and food neophobia still exert a strong influence on attitudes towards novel foods [16]. Previous studies have indicated that when consumers evaluate new food products, they consider not only safety and nutritional aspects but also perceived naturalness and cultural acceptability [17,18,19,20].

In Europe, from a historical and cultural context, protein consumption patterns differ considerably among countries. For instance, Northern European diets are traditionally rich in animal-based products such as fish and wild game, whereas plant-based protein has long been part of Southern European diets. This distinction can be traced back to the cultural influences of ancient Greece and Rome, where the consumption of meat was often regarded as a barbaric practice [21]. Furthermore, the widespread adherence to the Mediterranean diet has promoted high intake of vegetables, fruits and cereals [22].

Consumer attitudes towards alternative protein options are shaped by a combination of psychological, sociocultural and product-specific factors [23]. Although the potential of food innovation to promote sustainable consumption is widely acknowledged, public acceptance remains inconsistent, particularly regarding trust in food systems and alignment with personal values [16,24]. Recent Southern European research has begun to address this gap, highlighting how cultural attachment to traditional diets, perceived naturalness, and food neophobia influence acceptance of novel protein sources in Portugal [25,26], Spain [27,28,29,30], Italy [31,32,33,34,35], and Greece [36]. Nevertheless, such studies remain relatively limited compared with those conducted in northern and central Europe. Understanding how demographic, dietary and attitudinal factors shape the perception and acceptance of alternative proteins in these culturally distinct contexts is therefore essential to develop effective communication strategies and promote the incorporation of next-generation proteins into everyday diets [37].

This study addresses this gap by investigating consumer perceptions, attitudes and behaviours towards alternative protein sources in the Piedmont region of northwestern Italy, a setting with a rich gastronomic heritage and distinct cultural food identity. Specifically, we assessed demographic and dietary characteristics of participants, their consumption patterns and willingness to try different alternatives, as well as the perceived barriers, concerns and drivers influencing acceptance.

Based on previous literature, we hypothesised that:

(i) Younger individuals, women and participants reporting greater attention to their dietary choices would be more likely to consume or express willingness to try alternative protein sources;
(ii) Socio-demographic factors (particularly age, sex, and dietary habit) would also shape perceptions of product safety, nutritional comparability and sustainability;
(iii) Consumers of alternative proteins would express more positive attitudes and fewer concerns compared with non-consumers.

In addition, we examined purchasing habits and preferred modes of consumption in order to gain insights into the factors shaping the integration of alternative proteins into everyday diets.

## 2. Materials and Methods

### 2.1. Survey Design and Strategy

An online questionnaire was implemented through Google Forms© (Google LLC, Mountain View, CA, USA; Supplementary Material S1, File S1). The aim was to assess consumers’ perception about alternative protein sources, with regard to legume-based products (i.e., burgers), soy, tofu and tempeh, seitan, insect-based products, dried seaweeds and snails. The selection of these products was made on the basis of their ubiquity within the commercial sector; snails were chosen as a typical Piedmont product.

The questionnaire was structured in three main sections: the first section was dedicated to collecting sociodemographic data; the second section focused on dietary habits and the third section addressed food purchase behaviours, willingness to consult the nutritional labelling and the information sources used to consult dietary recommendations. Most questions incorporated closed multiple-choice answers and Likert-scale questions. The questionnaire was designed to be completed within 15 min and was to be conducted anonymously by adult individuals (aged ≥ 18 years) residing in Piedmont. Notwithstanding, a letter regarding the processing of personal data was provided with the questionnaire. In this letter, participants were informed about the objectives of the survey and were asked to authorise the use of their data in accordance with the stipulations of the General Data Protection Regulation (EU) 2016/679 [38] for the purposes of the survey.

The decision to focus on the Piedmont region was motivated by two factors. Firstly, the region’s specific socio-cultural characteristics render it a particularly interesting context for investigating the perception of alternative protein sources. Indeed, Piedmont is home to a global organisation that promotes food education, the preservation of traditional local foods, and sustainability in food production (Slow Food, [39,40]). Furthermore, the region hosts the University of Gastronomic Sciences (Pollenzo, Cuneo [41]), a prestigious institution specialising in the training of future professionals in the culinary arts. Secondly, the selection was driven by methodological considerations, namely the enhanced accessibility of diverse and heterogeneous population groups.

The questionnaire was developed by a multidisciplinary team of veterinarians, food safety experts, and nutritionists, based on existing literature on consumer perception of food products. The draft was reviewed for content validity by five independent experts and pilot-tested on a small group of volunteers (n = 15) to ensure clarity, consistency and comprehension before full deployment. No substantial modifications were required following the pilot test.

This survey adopted a non-probabilistic sampling strategy combining convenience and snowball sampling. The online questionnaire was distributed on 21 August 2023 through the institutional channels of the Istituto Zooprofilattico Sperimentale del Piemonte, Liguria e Valle d’Aosta, including its social networks, and remained open for completion until 31 October 2023. Moreover, consumers’ associations operating within the region were invited to share the survey among their members and relatives. In parallel, the gastronomic festivals scheduled within the survey period in Piedmont were identified, and face-to-face interviews were conducted with attending participants (Supplementary Materials S1, Table S1).

Because the questionnaire was distributed through open-access institutional channels and social networks, the total number of individuals reached could not be determined, and therefore participation rate could not be estimated. This approach may have introduced a degree of selection bias, as respondents familiar with digital platforms may have been more likely to participate. Likewise, participants recruited during gastronomic events, although approached randomly, may represent individuals particularly interested in food-related activities. Nevertheless, the combination of online and in-person recruitment helped broaden participation, resulting in responses from different municipalities across Piedmont and a heterogeneous demographic composition.

Given the exploratory nature of the study, no formal a priori sample size calculation was performed. However, the number of valid responses obtained (n = 627) exceeds the minimum sample size (≈384 respondents) estimated using Cochran’s formula [42] for a 95% confidence level, 5% margin of error, and a total adult population of 3,636,620 inhabitants in Piedmont (as of 1 January 2023 [43]).

### 2.2. Statistical Analysis

The collected data were stored in an Excel file and subsequently managed and analysed using Stata 17 (Stata.Corp, 2021, College Station, TX, USA). Descriptive statistics were reported as counts and percentages to summarise sociodemographic characteristics, dietary habits, consumption patterns and responses to individual questionnaire items. Given the ordinal nature and non-normal distribution of food consumption frequencies, the Kruskal–Wallis test was applied to compare frequency of food consumption across dietary groups (particularly between omnivores and flexitarians, the most representative and potentially comparable dietary groups in the study sample), to assess differences in purchase patterns and factors influencing the purchase of alternative proteins, and to evaluate the frequency of consulting nutrition labels. All tests were two-sided with α = 0.05, and analyses were conducted on complete cases.

To explore multivariate relationships, we performed multiple correspondence analysis (MCA) on categorical variables. Five separate MCA models were conducted to visualise: (i) patterns of consumption of alternative proteins according to sex, age strata and dietary habit; (ii) willingness to try plant-based and animal-derived options, according to sex and age strata; and (iii) attitudes towards alternative proteins in relation to sex, consumption status and willingness to try plant- or animal-based alternatives; (iv) perceived barriers to consumption, including sensory characteristics, cost and healthiness; and (v) concerns related to health, food safety, environmental sustainability, animal welfare, and product quality. All variables were treated as categorical, being converted into sets of indicator variables including: sex (female, male); age strata (18–29, 30–39, 40–49, 50–59, 60–69, ≥70 years); dietary habit (omnivorous, semi-vegetarian/flexitarian, vegetarian, vegan, carnivorous); consumption status of alternative proteins (consumer, non-consumer); willingness to try plant- or animal-based alternatives (willing, not willing); attitudes (curiosity, disgust, phobia, indifference); and perceived barriers and concerns—taste, texture, appearance, cost, healthiness, health and nutrition, product origin and safety, environmental sustainability, animal welfare and taste and quality—each coded as 1 when reported and 0 otherwise. The results were displayed on the first two dimensions, which accounted for the largest proportion of total inertia and provided the most interpretable visual representation of the associations among variables. The outputs of the models are presented in Supplementary Materials S2.

Logistic regression modelling was subsequently utilised to evaluate associations between demographic and behavioural factors and a series of binary outcomes. Two initial models assessed (i) self-perceived diet quality (healthy vs. unhealthy) and (ii) consumption of alternative protein products (consumer vs. non-consumer). For the first model, dietary group and self-reported health status were included as predictors; for the second, explanatory variables comprised sex, age strata, marital status, educational level, health status and dietary habit. Age, sex and dietary habit were retained a priori based on theoretical relevance, whereas the remaining covariates were evaluated using a backward stepwise approach with an entry criterion of *p* < 0.20. The Akaike Information Criterion (AIC) was used to identify the most parsimonious model, which included age, sex, marital status and dietary habit. Categories with very small frequencies (vegetarian, vegan, 70+ years) were excluded to avoid sparse-data bias.

Further logistic regression analyses were performed to investigate determinants of the willingness to try plant-based and animal-based alternative proteins. Both outcomes were coded as binary variables (1 = willing to try; 0 = not willing to try). Separate models were fitted for plant- and animal-based options, adjusting for age strata, sex, and dietary habit, while including consumption status (consumer vs. non-consumer) as an additional covariate.

Additional models were used to assess perceptions regarding: (i) the influence of cultural habits on consumption, (ii) whether alternative proteins are perceived as less controlled than traditional ones, (iii) the nutritional comparability between alternative and traditional protein sources, and (iv) their environmental sustainability. Each perception variable was treated as a binary variable (1 = “Yes”; 0 = “No”). Explanatory variables included age strata, sex, dietary habit and consumption status. Variable inclusion followed a stepwise selection procedure, retaining predictors with theoretical plausibility and the lowest AIC.

For all regression models, results were expressed as odds ratios (ORs) with 95% confidence intervals (CIs), and statistical significance was set at *p* < 0.05. No correction for multiple comparisons was applied, as each regression model investigated a distinct, theory-driven outcome. Statistical inference was therefore interpreted considering both effect sizes and 95% confidence intervals.

## 3. Results

**Figure 1:**
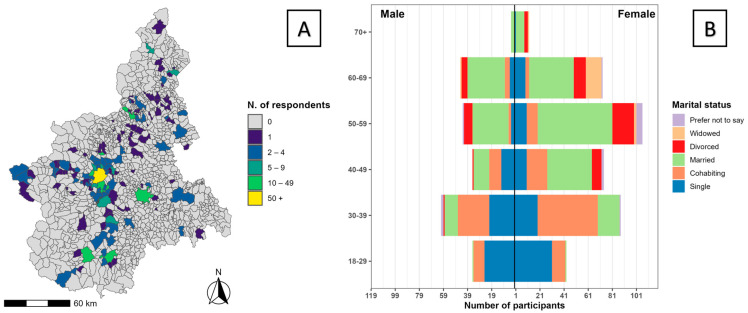
Spatial distribution of respondents and composition of the study sample. (**A**) Municipality-level choropleth of the number of survey respondents in Piedmont (n = 627), grouped into fixed classes: 0 (grey), 1, 2–4, 5–9, 10–49, and ≥50. (**B**) Population pyramid by age group and gender, with bars showing the number of participants and colours indicating marital status.

A total of 650 individuals participated in the survey. Of these, 15 declined consent and eight were excluded because information on their municipality of residence was either missing, uninformative, or outside Piedmont region. The final sample included 627 participants from 128 Piedmont municipalities, with 43.2% of responses collected in the city of Turin (Figure 1A). Women accounted for 63.3% of participants (sex not reported for 12 respondents, 1.9%), and the age distribution was relatively homogeneous across groups (∼20% each), except for those aged 70+, who represented only 2.4% of the sample (Figure 1B). Most participants reported at least holding a high school diploma or a higher education degree (97.3%; of which 45.1% held a bachelor’s or master’s degree). The 70.0% (n = 439) of the participants were employed full time, mainly in the medical/veterinary services, public administration, and education sectors.

Diet-related health conditions were reported by 22.7% (n = 142) of participants. The most common were hypertriglyceridemia (42.1%), blood pressure disorders (37.9%), and glycaemic disorders (16.4%), followed by food allergies or intolerances (9.3%). Other conditions, including overweight/obesity, gallstones, and gastric reflux, were reported less frequently (n = 12). Most respondents (85.8%) considered themselves to follow a “healthy diet”, defined as varied and balanced without excluding any food group. An omnivorous diet was most frequently declared (n = 484; 77.2%), followed by semi-vegetarian or flexitarian (n = 107; 17.1%). A low proportion of participants declared to be strictly vegetarian (n = 28; 4.4%), vegan (n = 6; 1.0%), or carnivorous (n = 2; 0.3%). The perception of a “healthy diet” did not differ by diet type (*p* = 0.219) or health status (*p* = 0.655).

**Figure 2:**
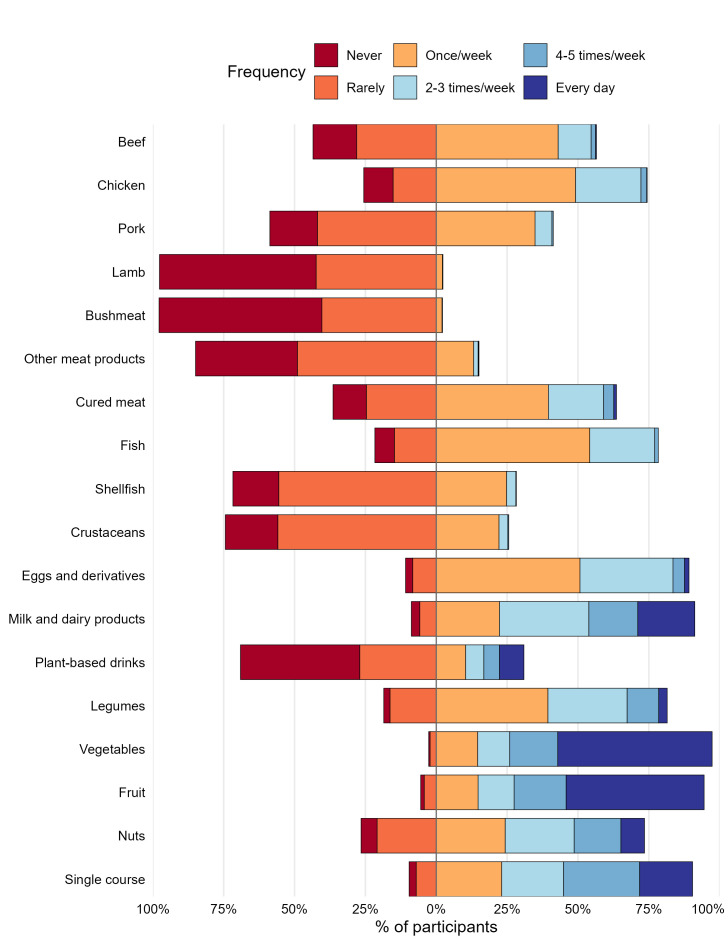
Patterns of respondent food intake by category and frequency (Never → Every day). Diverging stacked bars show the proportion of respondents reporting each intake frequency, centred at 0%: “Rarely” and “Never” to the left (below 0) and “Once/week” to “Every day” to the right (above 0). Single course includes the combination of pasta/cereals + protein + fat.

Figure 2 provides an overview of food consumption reported by participants. Overall, the consumption of plant-based products was found to be more prevalent than those of animal origin. Among animal-derived foods, milk and dairy products and eggs were the most commonly consumed, with 69.0% and 38.5% of respondents, respectively, reporting consumption on at least two days per week. For meat, chicken (74.4%) was the most frequently consumed, followed by veal (56.4%) and pork (41.3%), whereas lamb and bushmeat were rarely consumed (4.2%). Fruit and vegetables were widely consumed: only 2.7% and 5.8% of respondents reported never or rarely eating fruit and vegetables, respectively. Differences were observed according to diet groups. Semi-vegetarian/flexitarian respondents consumed legumes, nuts, and plant-based drinks (soy, rice, oat, almond, etc.) more frequently than omnivores (Kruskal–Wallis, *p* < 0.001). Conversely, omnivores consumed milk and dairy products more often, although 58.9% of semi-vegetarian/flexitarian respondents also reported intake at least twice per week. No differences were observed for egg, fruit, or vegetable consumption (*p* > 0.05).

**Figure 3:**
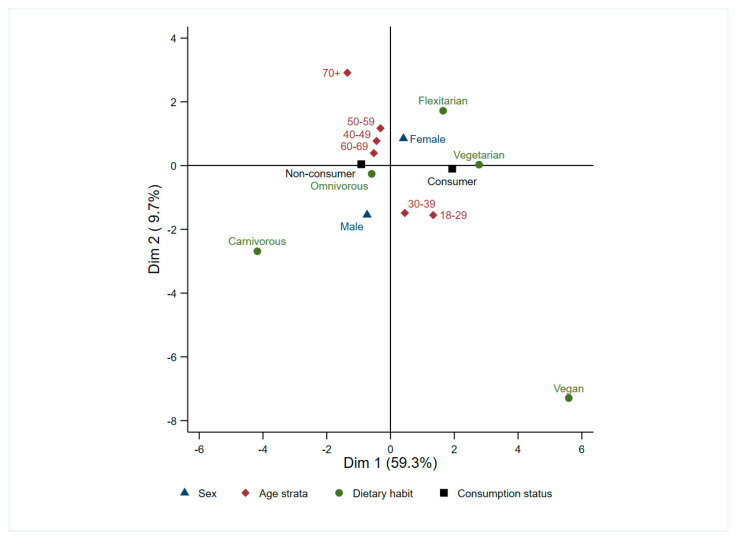
Multiple correspondence analysis (MCA) of sex, age strata, dietary habit and consumption status of alternative protein products. Dimension 1 (59.3% inertia) captures the main consumer vs. non-consumer contrast; Dimension 2 (9.7%) adds a separation among sex, age and dietary groups. Points represent category centroids; proximity indicates categories that tend to co-occur in respondents’ profiles.

Overall, 32.7% (n = 205) of respondents reported consuming alternative protein products. Both descriptive and multivariate analyses indicated that age, sex, and dietary habit influenced consumption patterns (MCA Dim 1= 59.3%; Figure 3). In particular, women (n = 146; 36.3%) were more likely than men (n = 54; 24.2%) to report intake and uptake seemed to decline with increasing age, with younger adults (18–39-year-olds) being the most frequent consumers (n = 106; 44.2%), whereas only 18.9% of those aged ≥ 40 years reported consumption. Consistent with dietary profiles, semi-vegetarian/flexitarian (n = 61; 57.0%), vegetarian (n = 21; 72.4%) and vegan (n = 7; 100%) respondents reported consumption more often than omnivores (n = 119; 24.3%), while none of the two strictly carnivorous respondents reported consuming alternative proteins.

These associations were further confirmed by logistic regression analysis (n = 549), showing that younger age, female sex, and semi-vegetarian/flexitarian dietary habits were independently associated with the consumption of alternative protein products (Table 1). Compared with respondents aged 18–29 years, those aged ≥ 30 years were less likely to consume such products, whereas men showed lower odds of consumption than females (OR = 0.49; 95% CI = 0.32–0.76). Semi-vegetarian/flexitarian respondents were markedly more likely to consume alternative proteins compared with omnivores (OR = 4.50; 95% CI = 2.79–7.24). Marital status was not associated with consumption.

**Figure 4:**
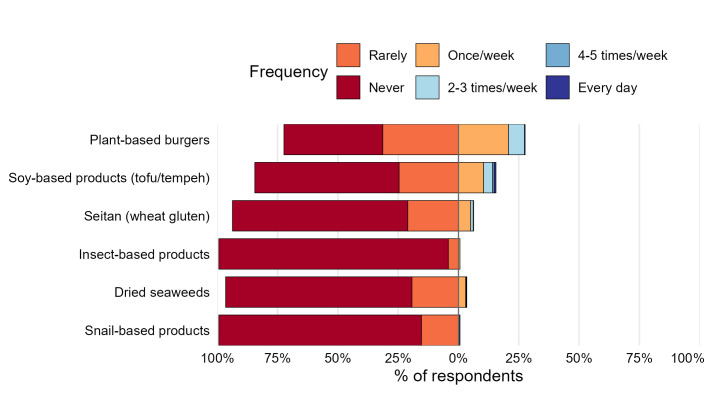
Patterns of respondent intake by alternative protein products and frequency (Never → Every day). Diverging stacked bars show the proportion of respondents reporting each intake frequency, centred at 0%: “Rarely” and “Never” to the left (below 0) and “Once/week” to “Every day” to the right (above 0).

Among consumers, 30.7% reported using these products exclusively as substitutes for meat products. Plant-based alternatives were by far the most popular alternatives (Figure 4), including plant-based burgers (84.9%), soy-based products, tofu, or tempeh (46.3%), seitan (18.5%), and algae-based products (10.2%). Only four participants (2.0%) declared consumption of insect- or snail-based products.

The consumption of alternative protein products did not appear to represent a barrier to social inclusion. Among consumers, 50.7% felt socially accepted and 27.8% had never considered the issue and only 16.6% of participants reported feeling judged.

**Figure 5:**
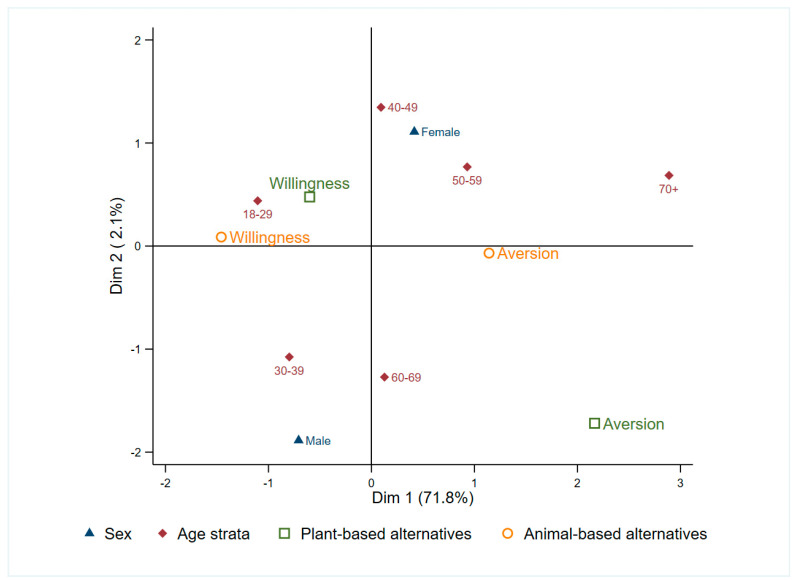
Multiple correspondence analysis (MCA) of willingness to try plant- and animal-based alternative protein products by sex and age strata. Dimension 1 (71.8% inertia) captures the main willingness contrast to try alternative options; Dimension 2 (2.1%) adds a separation among sex and age groups. Points represent category centroids; proximity indicates categories that tend to co-occur in respondents’ profiles.

Willingness to try plant-based proteins was markedly higher than for animal-derived alternatives. Overall, 92.8% of consumers and 72.7% of non-consumers were open to try plant-based alternatives, while willingness towards animal-derived alternatives (insect- or snail-derived products) dropped to 51.9% and 39.5%, respectively. We observed patterns of broader acceptance of plant-based products, with a clear age–sex divide for animal-based alternatives (MCA Dim 1 = 71.8% inertia; Figure 5): younger adults (18–39 years-olds) were generally more open to try both product categories, while willingness seemed to decline with age, especially for insect- and snail-based alternatives. Women and younger participants tended to express more positive attitudes towards plant-based options, whereas men showed more openness to animal-based ones. These patterns were confirmed statistically: willingness to try plant-based products was strongly linked to prior consumption experience (OR = 3.51; 95% CI = 1.90–6.49), and to a semi-vegetarian/flexitarian profile (OR = 1.59; 95% CI = 0.81–3.13), while older age tended to reduced willingness (Supplementary Materials S3). For animal-based alternatives, male respondents were more than twice as likely as females to express willingness (OR = 2.30; CI = 1.57–3.38), and previous consumption experience remained a major determinant (OR = 2.54; 95% CI = 1.64–3.92).

**Figure 6:**
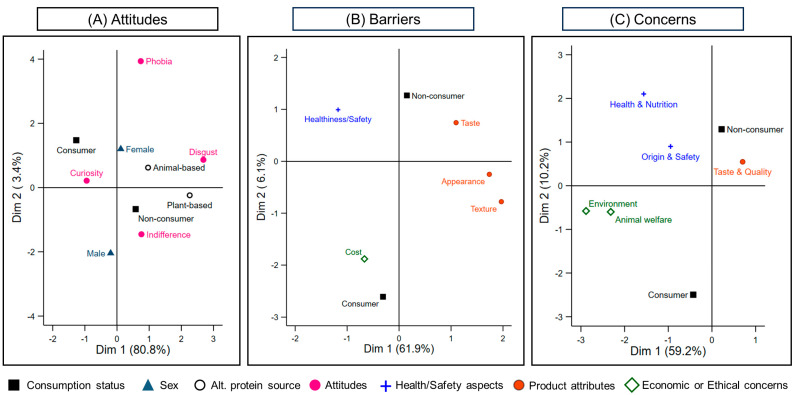
Multiple correspondence analyses (MCA) of attitudes (**A**), perceived barriers (**B**) and concerns (**C**) about alternative protein products. In (**A**) Dimension 1 (80.8%) captures the main contrasts among feeling characteristics; Dimension 2 (3.4%) adds a separation among sexes. In (**B**) Dimension 1 (61.9%) captures the main contrasts between sensory and economic and health related factors; Dimension 2 (6.1%) adds a separation based on sex and consumer’s status. In (**C**) Dimension 1 (59.2% inertia) captures the main ethical–environmental and product characteristic contrast; Dimension 2 (10.2%) adds a separation among consumer’s status. Points represent category centroids; proximity indicates categories that tend to co-occur in respondents’ profiles.

Attitudes towards alternative proteins were varied and were chiefly structured by consumption status (MCA Dim 1 = 80.8% inertia; Figure 6A). Overall, 59.2% of respondents expressed curiosity and interest, 24.1% declared indifference, 17.7% reported disgust, and 18 participants (2.9%) indicated phobia (Supplementary Material S1, Table S2). Regular consumers of alternative protein products were more likely to express curiosity and interest (81.5% vs. 48.3% of non-consumers), whereas non-consumers more often declared indifference (29.4% vs. 13.2% of consumers). Feelings of disgust and phobia were predominantly reported by non-consumers (20.1% and 2.9%, respectively) and were mainly directed at animal-based alternatives; among women aversion to animal-based products was particularly marked (76.6%; Figure 6A). Notably, none of the 26 consumers of plant-based protein alternatives reported consuming animal-based alternatives and 12 respondents, regardless of consumer status, explicitly rejected insects, snails, or other animal-derived products.

Cultural habits did not appear to influence consumption of alternative protein products, with 17.1% of consumers and 27.5% of non-consumers reporting this factor as determinant to do so (Table 2). However, three broad domains of barriers were identified to drive the consumption of such products (MCA Dim 1 = 61.9% inertia; Figure 6B). These included sensory attributes (appearance, texture, taste), as reported by 64.2% of respondents, the product healthiness and safety (29.8%) and its cost (28.2%). Within the sensory domain, taste was the most frequent barrier cited by non-consumers (52.9%), followed by appearance (39.1%) and texture (35.1%). Among consumers, the same barriers were common—taste (39.0%), appearance (38.5%) and texture (37.6%)—but generally at lower or similar levels (Supplementary Material S1, Table S2; Figure 6B). Cost emerged as a relatively greater concern among consumers (33.7% vs. 25.6% in non-consumers), whereas healthiness and safety were reported by 31.8% of non-consumers and 25.9% of consumers (Supplementary Material S1, Table S2).

The most frequently cited concerns about alternative proteins were taste and product quality (59.0%), and product origin and safety (52.3%). While both consumers and non-consumers commonly reported taste and product quality (Supplementary Material S1, Table S2), sensory issues characterised non-consumers more strongly, whereas consumers’ concerns were broader and tended to emphasise safety/health and ethical–environmental aspects (MCA Dim 1 = 59.2% inertia). Consistently, consumers more often report concerns about impacts on health and nutrition (26.8% vs. 18.0%), animal welfare (30.2% vs. 20.1%) and environmental impact (22.9% vs. 17.5%), whereas concerns about product origin and safety were slightly more reported by non-consumers (54.0% vs. 48.8%).

A minority of respondents (12.4%) considered alternative proteins to be less controlled than traditional ones, a perception more common among non-consumers (15.6%) than consumers (5.9%), although almost half of the participants (46.6%) declared lack of knowledge on this aspect (Table 2). Age also influenced this view, with respondents aged 40–49 years exhibiting higher odds compared with younger adults (OR = 3.23; 95% CI = 1.06–9.85). No significant effects were otherwise observed for sex or dietary habits (Supplementary Materials S3). Regarding nutritional comparability, 43.7% of participants viewed alternative and traditional protein sources as equivalent. Consumers (OR = 1.66; 95% CI = 1.03–2.67), men (OR = 1.61; 95% CI = 1.06–2.46), flexitarian/semi-vegetarian participants (OR = 3.14; 95% CI = 1.58–6.22) and older adults aged 60–69 years (OR = 2.63; 95% CI = 1.24–5.56) were more likely to share this view (Supplementary Materials S3). A similar pattern emerged for perceptions of environmental sustainability: 30.9% of respondents considered alternative and traditional proteins comparable in this regard, with a higher proportion among consumers (38.4%; OR = 1.63, 95% CI = 1.01–2.61). Male respondents were twice as likely as females to share this view (OR = 2.03; 95% CI = 1.30–3.17) and flexitarian/semi-vegetarian participants exhibited a marginal tendency toward higher agreement (OR = 1.63; 95 CI = 0.93–2.85).

Regarding food purchase and consumption habits, supermarkets represented the main procurement channel for all food (60.9%), and also for alternative proteins (64.9%). However, markets (13.9% vs. 5.4%), small shops (19.5% vs. 8.0%), and online purchases (8.8% vs. 1.8%) were reported more frequently among consumers of alternative proteins (*p* < 0.01). The main drivers of food purchase were taste (76.6%), nutritional (73.5%), and safety (73.7%) aspects, whereas novelty (25.2%) and price (16.7%) were considered less decisive. By contrast, for alternative protein products, price appeared to more influential, with 84.3% of consumers reporting moderate to high impact, compared with 36.7% of non-consumers who declared little or no influence (Kruskal–Wallis, *p* < 0.01). Product origin and quality were also considered relevant (59.8% of respondents). Notably, 29.9% of non-consumers stated they would still prefer traditional proteins even if alternative products were cheaper (*p* < 0.001). Most participants preferred consuming alternative proteins at home (56.1%) and combined with other ingredients (77.5%), patterns similarly observed among consumers and non-consumer participants (Table 2).

Nutritional labels were frequently consulted by 75.1% of respondents, with higher frequency among alternative protein consumers compared with non-consumers (Kruskal–Wallis, *p* < 0.001). Indeed, 28.9% of non-consumers declared rarely or never consulting labels, compared with 16.6% among consumers. Carbohydrates, proteins, and fats were the most frequently checked nutritional values. Finally, the main reported sources of information on nutrition were media (32.2%) and nutritionists (29.5%).

## 4. Discussion

In our survey, one-third of respondents reported consuming alternative protein products, with uptake particularly high among women, younger individuals and those following flexitarian diets. Plant-based alternatives were by far the most popular protein options, while insect- and snail-based products were rarely consumed. Willingness to try alternative proteins was generally high for plant-based options but markedly lower for animal-derived alternatives, particularly among older respondents and women. These findings confirm that acceptance of novel protein sources is not evenly distributed across the population, but is shaped by a combination of sociodemographic, cultural and dietary factors. This heterogeneity underscores the influence of deeply rooted Mediterranean dietary traditions, in which food choices are strongly linked to identity, heritage and symbolic meanings associated with naturalness and culinary familiarity [44,45].

Food production exerts mounting pressure on climate, biodiversity and natural resources [46,47]. In this context, dietary shifts towards reduced meat consumption and greater reliance on legumes, nuts and other plant proteins are recommended [4,48,49]. Our results partly reflect this transition: although most respondents still identified as omnivores, they reported higher consumption of plant-derived foods than of animal-based ones, suggesting a gradual move towards more plant-forward diets. In Southern Europe, this trend can be seen as a reinterpretation of the Mediterranean dietary model, where traditional plant-based ingredients are being reframed as modern, sustainable choices rather than dietary restrictions.

Engagement with alternative proteins in our sample was patterned by gender, age and dietary style. Women and younger respondents were more likely to consume these products, and flexitarians reported greater intake of legumes, nuts and plant-based drinks than omnivores, consistent with diversification of protein sources under reduced-meat regimes. This observation reflects the defining features of flexitarian diets, which emphasise legumes, nuts, soy and other plant-derived proteins as substitutes for meat [50,51]. These trends align with recent studies showing higher acceptance of plant-based alternatives among women and younger consumers, while men are more open to consuming animal-derived alternatives [37,52,53,54,55,56]. This divergence likely reflects both biological and cultural factors [57]: men generally consume greater quantities of meat than women, a pattern deeply rooted in traditional gender norms and symbolic associations between meat, strength and masculinity [58,59,60,61]. Conversely, plant-based eating is often culturally linked with care, health consciousness and environmental concern. In this context, women’s greater engagement with plant-based alternatives may reflect both ethical and health-oriented motivations, whereas men’s higher openness to animal-derived options may be shaped by continuity with traditional consumption habits and cultural symbolism surrounding meat [62]. Such gendered meanings of food remain particularly salient in Mediterranean societies, where culinary practices are intertwined with identity and social roles. The decline in consumption with age observed in our survey is partly consistent with Moruzzo et al. [63], who reported that older consumers are less receptive to insect-based proteins, although our findings indicate that this trend may extend more broadly to other alternative protein sources.

Across Europe, plant-based alternatives consistently enjoy greater familiarity and acceptance than insect- or snail-derived products, a pattern we also observed. A recent multi-country study has drawn parallels between the anticipated levels of taste, healthiness and environmental friendliness of fourteen distinct protein sources, showing legumes and common plant protein as front-runners, with insects ranked among the least acceptable [63]. Additional European studies similarly attribute low insect uptake to disgust, lack of tradition, and safety concerns [28,37,64,65,66,67]. This scenario is consistent with European eating habits in general, given that the insect market has been recently introduced in Europe (Regulation (EU) 2021/882 [68]). Moreover, insects, in particular, are often perceived as a source of contaminants and, consequently, associated with health risks, which reinforces negative attitudes [69,70,71]. By contrast, plant-based substitutes benefit from familiarity and alignment with existing dietary patterns, which may explain their broader acceptability. As highlighted by Pronk et al. [37] and Onwezen et al. [55], legumes and plant-based proteins rank highest in consumer preference compared with algae, insects, or cultured meat. Our data therefore suggest that plant-based proteins currently represent the most promising entry point for accelerating the protein transition in Western contexts, while acceptance of animal-derived alternatives remains constrained by cultural and psychological barriers. In Southern European cultures, where food heritage, artisanal production and ingredient provenance are highly valued [72], overcoming this barrier requires not only technological innovation but also cultural adaptation—framing novel proteins within local gastronomic narratives rather than as replacements for traditional foods.

Attitudinal responses varied from curiosity among regular consumers to indifference or disgust among non-consumers in this study, with a small minority reporting phobic reactions. These findings emphasise the importance of familiarity and positive tasting experiences for improving acceptance of novel foods [73,74]. Conversely, disgust sensitivity and perceived “unnaturalness” diminish acceptance [37,75]. Evidence also shows gendered patterns in disgust towards novel foods, especially those derived from insects, with women often reporting higher aversion [28,64,65,76]. These findings indicate that strategies aimed at enhancing familiarity—particularly through direct product experience—may be more effective than relying solely on information campaigns. In Mediterranean contexts, where collective eating and sensory enjoyment are central to food culture [45], acceptance strategies should appeal to taste, pleasure and tradition, not only to environmental or ethical arguments.

Sensory and intrinsic product attributes (especially taste and texture) are the most salient barriers to acceptance in our survey. Taste was most frequently reported, particularly by non-consumers, confirming that organoleptic qualities remain central to consumer decision-making [73,77]. Concerns regarding product origin, safety and nutritional equivalence were also raised, although many respondents admitted they lacked sufficient knowledge to make an informed judgement. Views on sustainability were similarly divided: comparable proportions of participants considered alternative proteins equivalent, inferior, or non-comparable to conventional ones. This uncertainty echoes broader evidence showing perceptions of novel foods are often shaped by heuristics such as naturalness and by trust in the food system [16]. Moreover, price emerged as a critical constraint, particularly among regular consumers, suggesting that economic accessibility may remain a decisive factor in the expansion of alternative protein markets. Indeed, recent studies highlight that plant-based meat alternatives are, on average, significantly more expensive than conventional meat—about twice as costly as beef and several times more than pork or chicken [78]. Even when prices drop, these products continue to carry a persistent premium, which may delay their competitiveness—though models suggest potential parity for certain products, such as burgers, within the near future [79,80]. From a policy perspective, incentives for local production and price equalisation mechanisms could accelerate market adoption, while industry stakeholders should prioritise flavour optimisation and integration of traditional ingredients (e.g., legumes, grains and herbs) typical of Mediterranean cuisines.

Perceptions were also influenced by social and cultural dimensions. Most respondents felt socially accepted in their dietary choices, indicating that the consumption of alternative proteins does not generally generate stigma. This may reflect the growing ethical and sustainability narratives that legitimise these products [55,81]. At the same time, respondents expressed a preference for consuming alternative proteins at home and in combination with other ingredients, rather than as stand-alone products in restaurants or street food settings. This aligns with earlier findings that consumers prefer to try alternative proteins in closer social contexts and when incorporated into familiar, processed formats such as snacks, soups, or burgers without visible insect parts [82,83]. These preferences indicate that consumers may be more willing to experiment with novel proteins when they can be incorporated into everyday meals. Evidence also indicates that contextual factors such as the social setting and the circumstances under which novel foods are consumed can meaningfully influence consumers’ willingness to try novel foods, with social and familiar contexts promoting greater openness [73,84,85,86]. From a practical standpoint, integrating alternative proteins, notably those of animal origin, into conventional Italian and European culinary preparations, and presenting them in familiar, processed forms rather than whole (visible) ones, may help normalise their consumption [87]. Framing these innovations within the Mediterranean diet, with its emphasis on local ingredients, biodiversity and balance, may further ease their cultural integration. Moreover, educational initiatives linking these innovations to Mediterranean sustainability values could reinforce public trust and support long-term dietary transition.

This study has some limitations. The sample was restricted to a single Italian region and skewed towards highly educated, professionally active individuals, which may limit generalisability to other populations. Reliance on self-reported data may also have introduced recall or social desirability biases. Despite these caveats, the study provides valuable insights into consumer perceptions of alternative proteins, offering a basis for future research and policy initiatives.

Overall, our findings reinforce that consumer acceptance of alternative proteins is shaped by a multifaceted interplay of sociodemographic, cultural, psychological and economic factors. The results underline that in Mediterranean societies, cultural continuity and culinary identity remain key determinants of food acceptance. Plant-based proteins appear best positioned to facilitate dietary transitions in the near term, while broader adoption of animal-derived alternatives will depend on overcoming sensory, safety and cultural barriers. Practically, this calls for communication strategies that emphasise the compatibility of alternative proteins with Mediterranean eating traditions, leveraging cultural pride and local gastronomy rather than framing these products solely as futuristic or technological solutions.

## Figures and Tables

**Table 1:** Multivariate logistic regression model for factors associated with the consumption of alternative protein products among adults (n = 549) in Piedmont, Italy.

Covariate	Category	OR	95% CI	*p*-Value
**Sex**	Female	1 (Ref.)	—	—
	Male	**0.49**	**0.32–0.76**	**0.002**
**Age strata (years)**	18–29	1 (Ref.)	—	—
	30–39	**0.39**	**0.20–0.78**	**0.007**
	40–49	**0.23**	**0.10–0.50**	**<0.001**
	50–59	**0.34**	**0.15–0.75**	**0.010**
	60–69	**0.23**	**0.10–0.53**	**0.001**
**Dietary habit**	Omnivorous	1 (Ref.)	—	—
	Semi-vegetarian/Flexitarian	**4.50**	**2.79–7.24**	**<0.001**
**Marital status**	Single	1 (Ref.)	—	—
	Cohabiting	1.59	0.88–2.88	0.123
	Married	1.10	0.59–2.06	0.763
	Divorced/Separated	1.23	0.50–3.01	0.649
	Widowed	0.66	0.15–2.98	0.590
**Model fit:** LR χ^2^(10) = 76.93; *p* < 0.001; Pseudo R^2^ = 0.115; AIC = 614.6.				

Note: Estimates in bold are statistically significant. Categories with very small frequencies in the covariates age strata (70+ years) and dietary habit (vegetarian, vegan) were excluded from the model to avoid sparse-data bias. The reference categories (Ref., OR = 1) were female (sex), 18–29 years (age strata), omnivorous (dietary habit), and single (marital status).

**Table 2:** Perceptions and modes of consumption regarding alternative proteins stratified by consumers and non-consumers.

	Consumers (n = 205)	Non-Consumers (n = 422)	Odds Ratio (OR) [95% CI]	*p*-Value
Do cultural habits influence your choice to consume alternative protein sources?:				
Yes, they are decisive	35 (17.1)	116 (27.5)	–	
Partially, I am open to new habits	105 (51.2)	190 (45.0)	–	
No	65 (31.7)	107 (25.4)	–	
Do you consider alternative proteins less controlled than traditional ones?:				
Yes	12 (5.9)	66 (15.6)	0.32 [0.16–0.66]	0.002
No	109 (53.2)	148 (35.1)	–	
Don’t know	84 (41.0)	208 (49.3)	–	
Do you consider alternative proteins fully comparable to traditional ones from a nutritional perspective?:				
Yes	125 (61.0)	149 (35.3)	1.66 [1.03–2.67]	0.038
No	34 (16.6)	124 (29.4)	–	
Don’t know	46 (22.4)	149 (35.3)	–	
Do you consider alternative proteins fully comparable to traditional ones from an environmental sustainability perspective?:				
Yes	83 (40.5)	118 (28.0)	1.63 [1.01–2.61]	0.043
No	69 (33.6)	142 (33.6)	–	
Don’t know	53 (25.9)	162 (38.4)	–	
Modes of consumption of alternative proteins:				
In combination with other ingredients	149 (72.7)	337 (79.9)	–	
Product consumed as such	56 (27.3)	85 (20.1)	–	
At restaurants	31 (15.1)	59 (14.0)	–	
At home	133 (64.9)	219 (51.9)	–	
Street food	11 (5.4)	16 (3.8)	–	
Themed events	20 (9.8)	38 (9.0)	–	

Note: the non-consumer category was considered as the reference group (OR = 1.00).

## Data Availability

The raw data supporting the conclusions of this article will be made available by the authors on request.

## Supplementary Materials

The following supporting information can be downloaded at: https://www.mdpi.com/article/10.3390/foods14213727/s1. Supplementary Material S1: File S1: Questionnaire; Table S1: Gastronomic festivals in Piedmont in which face-to-face interviews were conducted. Table S2. Attitudes, perceived barriers and concerns regarding alternative protein products by consumption status. Supplementary Material S2: Results of the Multiple Correspondence Analyses performed to explore associations between demographic, dietary, and attitudinal variables. The analyses include the following dependent domains: Table S3: Consumption of alternative protein products; Table S4: Willingness to try plant-based alternatives and animal-based (insect/snail-derived) alternatives; Table S5: Attitudes towards alternative protein options; Table S6: Perceived barriers and drivers influencing consumption; Table S7: Perceived concerns toward alternative protein products. Supplementary Materials S3: Complete regression outputs for the logistic models assessing demographic and behavioural determinants of key binary outcomes: Table S8: Model 1: Self-perceived diet quality (healthy vs. unhealthy); Table S9: Model 2: Consumption of alternative protein products (consumer vs. non-consumer); Table S10: Models 3A-B. Willingness to try plant- and animal-based (insect/snail-derived) alternatives; Table S11: Model 4: Perception that cultural habits influence alternative options consumption; Table S12: Model 5: Perceptions about control of alternative proteins compared with traditional proteins: Table S13: Model 6: Perceptions about nutritional comparability of alternative protein options compared with traditional proteins: Table S14: Model 7: Perceptions about environmental sustainability of alternative proteins compared with traditional ones.
