# Supplementary material for: From Curiosity to Consumption: Consumer Attitudes Toward Alternative Proteins in Northwestern Italy
Source: Foods. 2025 Oct 30;14(21):3727. doi: 10.3390/foods14213727 (PMC12608852; doi:10.3390/foods14213727)
Supplement: Supplementary file 1 [file foods-14-03727-s001.zip › Supplementary Material S1/File S1-Questionaire.pdf]

Novel Food: Alternative Protein Sources

Do you consume alternative protein sources?

Rationale and Objective of the Questionnaire

The consumption of animal-derived foods by humans has a long historical precedent, dating back a minimum of 5 million years. Such consumption has been subject to variation over the course of several decades, with fluctuations attributable to economic factors, social circumstances, and the transition between periods of war and peace. In more recent times, the rise of sustainability and environmental awareness has also exerted an influence on dietary habits and food production chains.

The aim of this survey is to gather information on the consumption of alternative protein sources in comparison to conventional ones, the willingness to consume products such as insects, snails, and legumes (chickpeas, beans, lentils), and the consumers' attention to the information provided on the nutrition label

In the following questions, the term 'alternative protein sources' refers to foods with a high protein content other than the mammalian meat or fish to which people are accustomed: namely, snails, insects, and plant-based proteins. The term 'nutrition label' refers to the mandatory label that provides consumers with valid information on the energy value and content of nutrients such as proteins, fats, carbohydrates, salt, fibre, vitamins, and minerals present in packaged products.

The questionnaire is anonymous and should take no more than 15 minutes to complete. Your participation in this study would be greatly appreciated, as your input would contribute valuable data to the project. The processing of data will be conducted anonymously and for the purposes of scientific research, in accordance with the provisions of the General Data Protection Regulation (Reg. EU 679/2016).

In the following sections, questions with * are mandatory.

Informed Consent and Authorisation for Data Use

I have read the objectives of the study, I voluntarily decide to participate in this questionnaire and I authorise the use of the data provided herein for the scientific purpose of the study. *

Yes
No

Demographics

1. Which age group do you belong to? *
18–29
30–39
40–49
50–59
60–69
70+

2. Sex *
Male
Female
Prefer not to say

3. What is your marital status? *
Single
Cohabiting
Married
Widowed
Separated
Divorced
Prefer not to say

4. What is your highest level of education? *
Primary education
Lower secondary education
Upper secondary education
Bachelor's/Master's/Single cycle degree
PhD/Postgraduate/Master's

5. Which category best represents your current work situation? *
Student
Grant holder/Intern
Precarious worker
Full-time employee
Part-time employee
Unemployed, seeking work
Unemployed, not seeking work
Retired
Other

6. Please indicate your professional field *
(Agriculture, Environment, Administration, Arts, Commerce, Media, Education, Armed forces, HR, Publishing, Retail, Food industry, IT, Engineering, Fashion, Non-profit, Public administration, Advertising, Catering, Healthcare, Social services, Sports, Telecommunications, Transport, Tourism, Cleaning services, etc.)

7. Please indicate your Province of residence in Piedmont *

8. Please indicate your Municipality of residence *

9. Nationality *

Health and Dietary Habits

10. Do you suffer from any health condition related to diet? (e.g. diabetes, high blood pressure, high cholesterol, or similar disorders) *
Yes
No

11. If yes, which?

12. Do you consider your diet to be healthy (understood as a varied and balanced diet complete with all essential elements without excluding any category of foodstuffs)? *
Yes
No
I don’t know

13. How would you define the type of diet you mainly follow? *
Vegetarian (“I don't eat meat or fish, but I still consume other animal products”)
Omnivorous (“I consume a wide variety of foods, including meat, eggs, milk and dairy products, fish and seafood, as well as cereals, fruit, vegetables and legumes”)
Semi-vegetarian / Flexitarian (“I mainly consume foods of plant origin, and I only occasionally eat foods of animal origin”)
Vegan (“I do not consume any animal products”)
Other

14. How often do you consume the following products? *

	Never	Almost never	Once a week	2-3 times a week	4-5 times a week	Every day
Veal						
Chicken						
Pork						
Lamb						

Game meat						
Other meats (e.g., horse, rabbit, duck, turkey)						
Cured meats and cold cuts						
Fish						
Shellfish (clams, mussels, octopus, cuttlefish, squid)						
Shellfish (shrimp, crab, lobster, etc.)						
Eggs						
Dairy products (milk, cheese, yoghurt)						
Plant-based drinks (soy, rice, oat, almond, etc.)						
Legumes (chickpeas, beans, peas, broad beans, lentils, etc.)						
Vegetables						
Fruit						
Dried fruit (walnuts, almonds, hazelnuts, etc.)						
Pasta dishes, risottos, omelettes, stir-fries based on vegetables/legumes/meat alternatives						

	Never	Almost never	Once a week	2-3 times a week	4-5 times a week	Every day
Plant-based burger (legume-based or cereal-based)						
Soy, tofu, tempeh						
Seitan						
Insect-based products						
Dried seaweed						
Snails						

15. Do you feel socially accepted when you choose to consume alternative protein foods instead of those of animal origin? *

Yes, I feel accepted and supported

No, I feel judged or marginalised
I have never reflected on this aspect
I do not consume this type of product

16. If you have never consumed alternative protein products, if given the opportunity, how curious would you be to try them? *

	I would never try them	I don't know	I would try them
Plant-based burger (legume-based or cereal-based)			
Soy, tofu, tempeh			
Seitan			
Insect-based products			
Dried seaweed			
Snails			

17. What do you feel towards alternative protein sources? *
Disgust
Fear
Indifference
Curiosity/interest
Other

18. Do you think cultural habits influence your choice to consume alternative protein products? *
Yes, my cultural habits are decisive
Partly, I am open to new habits
No, cultural habits do not influence my choice

19. What do you consider to be the main limitations to the consumption of alternative protein sources? *
Taste
Appearance
Texture
Uncertainty about their safety
Cost
Other

20. What are your main concerns about the use of alternative protein products? (Select all relevant options) *
Health and nutrition concerns
Origin and safety concerns
Environmental impact concerns
Animal welfare concerns
Taste and quality concerns
Other

21. In your opinion, are alternative protein sources less controlled than traditional protein sources (e.g. eggs, meat, etc.)? *
Yes
No
I don't know

22. In your opinion, which alternative protein source listed below contains the highest amount of protein? *
Insects and derivatives
Snails and derivatives
Dried seaweed
Legume-based products (soy, chickpeas, lentils…)

23. Would you prefer to consume an alternative protein source of: *
Plant origin
Animal origin
None of the above
I don't know

24. How would you prefer to consume alternative protein sources? *
As an ingredient combined with others
As a stand-alone product

25. Do you consider alternative protein sources to be fully comparable to traditional ones (meat, fish, etc.) from a nutritional point of view? *
Yes
No
I don't know

26. Do you consider alternative protein sources to be fully comparable to traditional ones (meat, fish, etc.) from an environmental sustainability point of view? *
Yes
No
I don't know

Purchasing Habits

27. How much do the following aspects influence your purchase of a food product? (1 = little, 5 = a lot) *

	1	2	3	4	5
Price					
Flavour					
Appearance					
Health and nutrients					
Country of origin					
Food safety					
Familiarity (meaning knowledge of the food)					
Convenience					
Animal welfare					
Production method (e.g. organic, free-range)					
Environmental impact					
Dietary tolerance/restrictions					
Novelty					

28. What is your usual purchasing channel? *
Hypermarket
Supermarket
Small shop
Open-air market
Online
Other

29. If you already consume alternative protein sources, what is your usual purchasing channel for these products? *
Hypermarket
Supermarket
Small shop
Open-air market
Restaurant
Online
Other

30. How much does price influence your decision to purchase alternative protein products? *
A lot, price is a decisive factor

Moderately, but I am willing to pay more for more sustainable options
A little, I am willing to spend more for higher quality products
Not at all, it does not influence my decision

31. If alternative protein products were cheaper than traditional options, would you be more likely to try them? *
Yes, I would choose the cheaper options
It depends on the quality and origin of the products
No, I still prefer traditional protein sources

32. Where would you prefer to consume alternative protein sources? *
Restaurants
At home
Street food
Themed event dinner
None of the above

33. How often do you read nutrition labels (information on energy value, amounts of fats, saturated fats, carbohydrates, sugars, proteins, salt, fibre, minerals, vitamins)? *
Always
Often
Rarely
Never

34. Which nutrition value(s) do you consider most important? *
Proteins
Sugars
Energy
Fats
Salt
Carbohydrates

35. If you are not a regular consumer of alternative protein sources, but were curious to try them, would you read the nutrition label of the product in question? *
Yes
No

36. About the nutrition values of alternative protein foods, which would you pay most attention to? *
Proteins
Sugars
Energy
Fats

Salt
Carbohydrates
None of the above

37. What is your main source of information about nutrition? *
Media (TV, newspapers)
Nutritionist/dietitian
Doctor
Friends/Relatives
Institutions
Other

38. Who would you prefer to be informed by regarding alternative protein sources? *
Media (TV, newspapers)
Nutritionist/dietitian
Doctor
Friends/Relatives
Institutions
