# Supplementary material for: From Curiosity to Consumption: Consumer Attitudes Toward Alternative Proteins in Northwestern Italy
Source: Foods. 2025 Oct 30;14(21):3727. doi: 10.3390/foods14213727 (PMC12608852; doi:10.3390/foods14213727)
Supplement: Supplementary file 1 [file foods-14-03727-s001.zip › Supplementary Material S1/Table S1. Sagre.pdf]

Table S1. Gastronomic festivals in Piedmont in which face-to-face interviews were conducted.

Gastronomic festivals	Municipality	Province	Scheduled day
Donkey Stew Festival	Calliano Monferrato	Asti	August 25, 2023
Tigelle and fried gnocchi Festival	Rivarossa	Turin	August 25, 2023
Tomato Festival	Cambiano	Turin	September 3, 2023
Pepper Festival	Carmagnola	Turin	September 4, 2023
Festival delle Sagre	Asti	Asti	September 3, 2023
