# Supplementary material for: From Curiosity to Consumption: Consumer Attitudes Toward Alternative Proteins in Northwestern Italy
Source: Foods. 2025 Oct 30;14(21):3727. doi: 10.3390/foods14213727 (PMC12608852; doi:10.3390/foods14213727)
Supplement: Supplementary file 1 [file foods-14-03727-s001.zip › Supplementary Material S1/Table S2.pdf]

Table S2. Attitudes, perceived barriers and concerns regarding alternative protein products by consumption status.

	**Consumers (n= 205)**	**Non-consumers (n= 422)**
**Attitudes towards alternative protein sources**		
Curiosity/Interest	167 (81.5)	204 (48.3)
Disgust	26 (12.7)	85 (20.1)
Phobia	6 (2.9)	12 (2.8)
Indifference	27 (13.2)	124 (29.4)
**Which aspects represent the main barriers to consuming alternative proteins?**		
Taste	80 (39.0)	220 (52.1)
Appearance	79 (38.5)	165 (39.1)
Texture	77 (37.6)	148 (35.1)
Product healthiness/safety	53 (25.9)	134 (31.8)
Cost	69 (33.7)	108 (25.6)
**What are your main concerns regarding alternative proteins?**	%	%
Impact on health and nutrition	26.8	18.0
Animal welfare	30.2	20.1
Environmental impact	22.9	17.5
Origin and product safety	48.8	54.0
Taste and product quality	63.0	50.7
